# Molecular Dynamics Approaches Dissect Molecular Mechanisms Underlying Methylene Blue–Glycosaminoglycan Interactions

**DOI:** 10.3390/molecules27092654

**Published:** 2022-04-20

**Authors:** Martyna Maszota-Zieleniak, Ferenc Zsila, Sergey A. Samsonov

**Affiliations:** 1 Faculty of Chemistry, University of Gdańsk, ul. Wita Stwosza, 63, 80-308 Gdańsk, Poland; m.maszota.zieleniak@gmail.com; 2 Institute of Materials and Environmental Chemistry, Research Centre for Natural Sciences, Hungarian Academy of Sciences, Magyar Tudósok Körútja 2, 1117 Budapest, Hungary; zsila.ferenc@ttk.hu

**Keywords:** methylene blue, glycosaminoglycan, sulfation pattern, molecular dynamics, oligomeric stacks

## Abstract

Glycosaminoglycans (GAGs) are a class of periodic anionic linear polysaccharides involved in a number of biologically relevant processes in the extracellular matrix via interactions with various types of molecules including proteins, peptides and small organic molecules. The metachromatic dye methylene blue (MB) is a GAG binding agent. This molecule possesses a tricyclic, monocationic phenothiazine ring system, while the terminal methyl groups attached to the nitrogen atoms bear the most positive charges of the cation and, therefore, represent potential binding sites for negatively charged GAGs. In this study, we rigorously explored molecular mechanisms underlying these interactions for several GAG types: heparin, heparan and chondroitin sulfates. We found that GAG–MB interactions are predominantly electrostatically driven, with the particularly important role of sulfate groups. MB oligomeric stack formation was favored in the presence of GAGs. Furthermore, the impact of MB binding on the conformation of GAGs was also evaluated. The novel results allow for better quantitative analytics of GAG composition in the studied biochemical systems using MB dye as a GAG-specific marker. Our data add to the knowledge on small molecule–GAG interactions and could be potentially useful for novel developments in drug design and putative disease therapies in which GAGs are involved.

## 1. Introduction

In recent decades, interest in the biochemical and biophysical properties of glycosaminoglycans (GAGs) has been steadily increasing [1]. These biopolymers composed of repeating disaccharide units of alternating uronic acid and hexosamine residues were originally considered to be merely the amorphous constituents of the extracellular matrix (ECM). However, GAGs have now been implicated as functionally relevant macromolecules in a variety of normal and pathological conditions, e.g., wound healing, tissue remodeling [2], cell cycle and signaling regulation [3], bacterial/viral infections [4,5], autoimmune/inflammatory disorders [6], cancer [7] and neurodegenerative diseases [8,9]. The family of GAGs consists of heparin (HP), heparan sulfate (HS), chondroitin sulfate (CS), dermatan sulfate, keratan sulfate and the non-sulfated hyaluronic acid. Except for hyaluronic acid, they are covalently attached to their respective core proteins forming proteoglycans present ubiquitously inside cells, on cell surfaces and in the ECM [1]. The unifying structural characteristics of these polysaccharides include linear, unbranched polymeric chains, polydispersity and a highly anionic nature due to a number of sulfate and carboxylate groups. Overall GAG structures consist of repeating disaccharide units composed of a hexosamine (*N*-acetyl-D-glucosamine or *N*-acetyl-D-galactosamine) and either uronic acid (glucuronic or iduronic acid) or a galactose ring. The structural diversity and conformational flexibility of their polymeric chains allow a multitude of non-covalent binding interactions with a vast number of bioactive protein and peptide partners, such as cytokines, growth factors, antibodies, enzymes, host-defense peptides, receptors and structural components of the ECM [10,11]. As well as these high-molecular weight substances, small, especially cationic organic molecules may also be GAG ligands such as alkaloids [12,13,14], pharmaceutical agents [15,16,17,18,19] and various aromatic dyes [20,21,22,23]. These interactions may have pharmacological importance resulting in the modulation of biological activities via perturbation of the GAG interactome [19,24]. GAG binding provokes peculiar changes in the absorption (metachromasia) [23,25,26], circular dichroism [12,16,17,18,20] and/or fluorescence spectra [27,28] of several heteroaromatic compounds including the phenazine, phenothiazine, thionine, cyanine and acridine family of dyes. The absorption and fluorescence spectral alterations served long ago as histological tools for the microscopic identification of tissue constituents [29] (Ribatti 2018). More recently, the great potential of synthetic dyes have also been recognized for quantifying heparin and related anticoagulant GAG derivatives in biological samples. Upon interaction with heparin, these colorimetric sensors exhibit a robust change in absorption signal intensity and/or peak position, which can be utilized in diverse bioanalytical applications [21,30,31].

**Figure 1:**
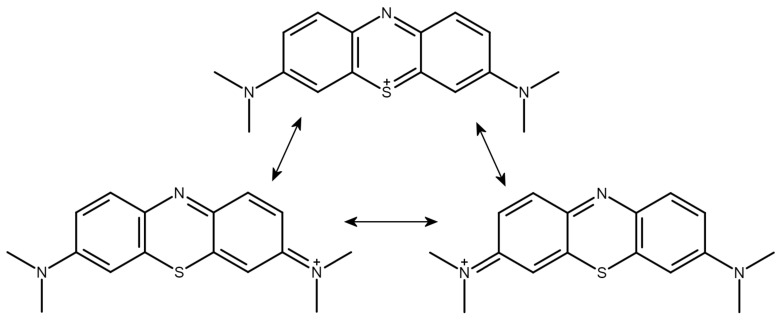
Conventional valence bond structures of the methylene blue cation with suggestions for the sites of the positive charge. Note that the most positive charges are located on the terminal methyl groups (see text).

The metachromatic dye methylene blue (MB), possessing a tricyclic, monocationic phenothiazine ring system, is a prototypical GAG ligand (Figure 1). It is to be noted that the formal positive charge of the dye is not placed on the sulfur atom but is distributed over a wide delocalized range outside the thiazine ring. The terminal methyl groups attached to the nitrogen atoms bear the most positive charges of the cation [32] (Luger et al. 2018).

MB binds to all major GAG types but with various stoichiometries and affinities [23,33,34,35,36,37,38,39]. It was the very first fully synthetic drug, used in medicine since 1891, with an indication to treat malaria. As an FDA approved agent, nowadays MB is administered in methemoglobinemia for the prevention of urinary tract infections in elderly patients, to alleviate ifosfamide-induced neurotoxicity and for the intraoperative visualization of nerves and endocrine glands [40]. Its methylated derivative, dimethylmethylene blue is the most widely employed colorimetric probe for sensing the sulfated GAG and proteoglycan content of biological samples [22,41,42].

However, in spite of the great deal of experimental data accumulated so far, molecular details of the underlying MB–GAG binding mechanisms are still poorly elucidated and thus remain a subject of continuing research. Although the importance of electrostatic attractions between cationic ligands and anionic GAG sites is well documented, in a recent study, Jia et al. concluded that less-sulfated heparinoids show a stronger MB binding ability than the more densely sulfated native HP [35]. Further on, they proposed the exclusive role of sulfate–MB ionic interactions in the stabilization of the complexes. On the other hand, Wang and co-workers suggested that as well as the sulfate residues, glucosamine rings give rise to the largest contribution to the spontaneity of MB binding events [36]. According to the hypothesis of Lawton and Phillips, the sulfate compared to the carboxylate group owns a larger effective ionic radius and thus binds the dye cations most weakly and allows them to self-associate most effectively [43]. Finally, Zhang et al. speculated that the binding of MB to chondroitin 4-sulfate induces the helical conversion of the originally random coil conformation of the GAG chains [37]. Taking these controversies and unsupported claims into account, we employed molecular dynamics simulations to obtain atomistic insights into MB–GAG interactions. Not only the structural and energetical features of the HP binding of MB were evaluated, but, to obtain a more complete picture, additional GAGs were also included in the simulations such as desulfated heparin, several types of heparan and chondroitin sulfates. The impact of dye binding on the conformation of GAG chains was also noted. All these data contribute to the practical knowledge on the use of MB as a GAG-specific dye in bioanalytical assays as well as to better understand its human interactome as a therapeutic drug.

## 2. Materials and Methods

### 2.1. Structures’ and Parameters’ Preparation

**Figure 2:**
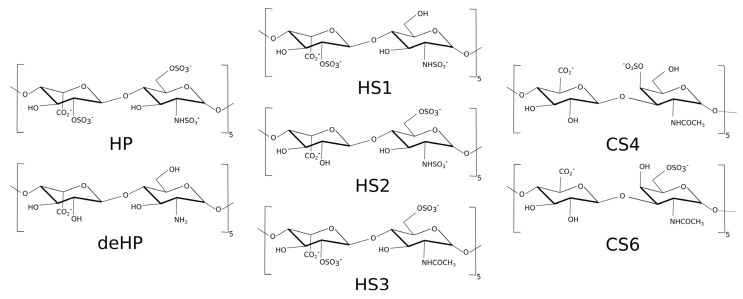
Three heparan sulfate (HS) dp10 structures used in this study.

The structure of methylene blue (MB) was built using Avogadro program [44]. Next, AM1-BCC [45] charge method was used for parametrization for general AMBER force field (GAFF) [46]. The structures of the heparin (HP), desulfated heparin (deHP), chondroitin sulfate-4 (CS4) and chondroitin sulfate-6 (CS6) with degree of polymerization 10 (dp10) were obtained in our previous work [47]. Three heparan sulfates (HS) dp10 structures were built from building blocks of the GAG monomeric units’ libraries [48]: (1) (GlcNS-IdoA(2S))_5_–HS1; (2) (GlcNS(6S)-IdoA)_5_–HS2; (3) (GlcNAc(6S)-IdoA(2S))_5_–HS3 (Figure 2). All these three HS oligosaccharides have a net charge of -3 per disaccharide periodic unit.

### 2.2. Molecular Dynamics

The AMBER20 program was used for all molecular dynamics (MD) simulations [49], for MB GAFF [46] force field parameters and for GAGs GLYCAM06 [50] force field parameters were used, respectively. Two sets of MD simulations were performed: (1) MB unbound: two and ten unbound drugs; (2) GAGs dp10 with ten MB molecules. All initial structures were built in the xLeap Amber20 program. The MB molecules were placed randomly around a GAG oligomer in different orientations. The periodic boundary conditions with TIP3P [51] cubic water box with 12 Å distance from solute atoms in each direction to the box wall were used. To neutralize the charge in the periodic box, Na^+^ counterions were added. First, an energy minimization, to remove close contacts between atoms, was performed, consisting of 3000 steps of steepest descent and 3000 steps of conjugate gradient. Then the complexes were heated up to 300 K for 10 ps in NVT ensemble and next simulations were performed in NPT ensemble until the density of solvent converged. As a final step, according to our earlier work made on similar systems [13,14,52], 100 ns of the production run simulation with SHAKE [53] algorithm was performed. The time integration step was 2 fs, the cutoff for non-bonded interactions was 8 Å, the Particle Mesh Ewald procedure was used [54]. The performed MD simulations run on the local cluster GPUs (Nvidia Tesla *K40d*) required approximately 40 h of wall-clock time per 100 ns. Obtained in the MD simulations, trajectories were analyzed in the CPPTRAJ program in AMBER Tools 20 [46]. All trajectories were visualized in VMD program [55], and the PyMOL [56] program was used for the production of figures.

### 2.3. Free Energy MM-GBSA Analysis and per Residue Decomposition

Molecular Mechanics Generalized Born Surface Area (MM-GBSA) was used for the energetic post-processing of the trajectories in AMBER 20 [49] program. The MM-GBSA analysis was performed using igb = 2 [57], for two unbound MB simulations and for the MB–GAG simulations. Frames where at least three MB molecules interact with GAGs were chosen for the analysis.

## 3. Results

**Figure 3:**
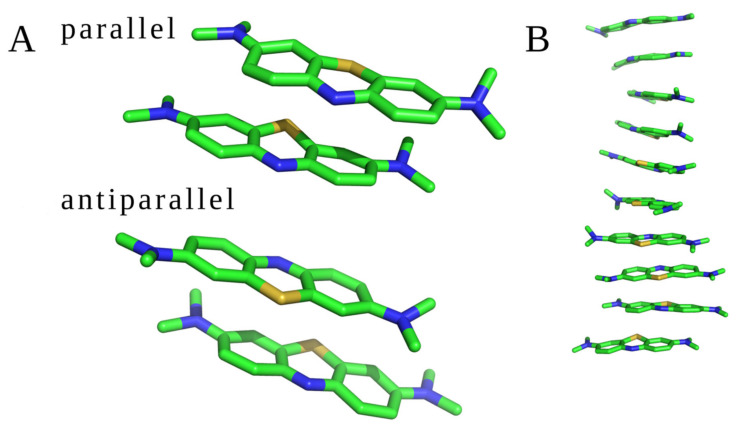
MB stacks observed in the MD simulations. (**A**) Two MB molecules in parallel and antiparallel orientations and (**B**) ten GAG-free MB (structures are displayed in stick representation; green—carbon atoms, blue—nitrogen atoms, and yellow—sulfur atoms).

MD studies were conducted for two different sets of the molecular systems: (1) two and ten unbound MB molecules; (2) MB molecules with GAGs dp10. All trajectories were visually inspected to check if the drugs interacted with each other and with GAGs. Two unbound MB molecules interacted with each other during the whole MD simulation establishing a dimeric complex in both parallel and antiparallel orientations (Figure 3A). During the MD simulation of ten unbound MB ligands, dye–dye interaction was also observed. The molecules formed oligomers containing from two to ten MB units in both parallel and antiparallel orientations (Figure 3B). Such stacks have been previously observed for other cationic aromatic dyes [13,14,20,52]. No other types of oligomeric configurations have been detected. In terms of the free energy of binding, the parallel and antiparallel stacks did not differ, yielding these configurations to be in equilibrium in the MD simulation. The average distance between two adjacent MB molecules, measured between the planes of the drug rings defined by aromatic carbons, was about 3.7 Å. Examples of the dimeric and oligomeric stacks are presented in Figure 3.

**Figure 4:**
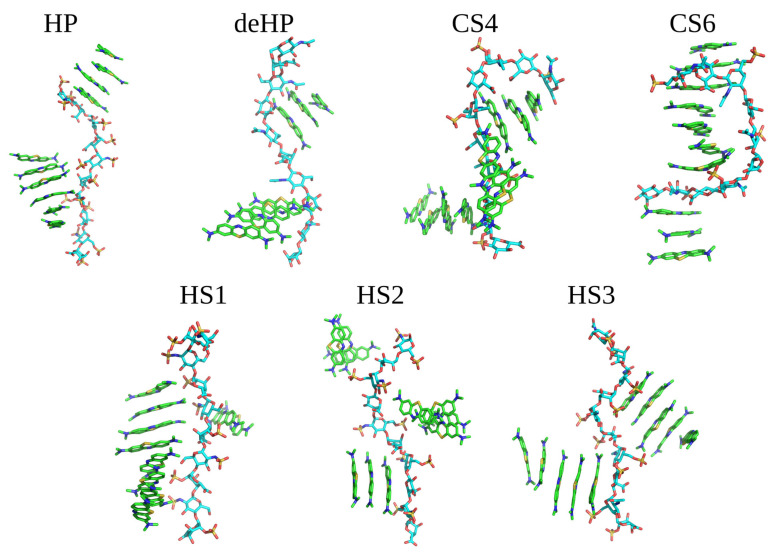
Examples of MB–GAG dp10 complex structures obtained in MD simulations (MB molecules are shown as in Figure 3; GAGs dp10: light blue—carbon atoms, red—oxygen atoms, blue—nitrogen atoms and yellow—sulfur atoms).

Dye stacking is promoted by the interactions with GAGs in terms of the speed of the stacks’ formation, but they are also formed anyway in the absence of GAGs. This finding is in line with the MD simulations of Wang et al. showing that HP increased the amount of MB dimer formation per 10 ns by 3.7-fold compared to the GAG-free state (Wang et al. 2018). The same average intrastack distance was observed when simulating 10 MB molecules in the presence of GAGs dp10 (Supplementary Material, Figure S1). For all analyzed MB–GAG dp10 complexes, the mechanism of the complex formation was similar: first, drug molecules formed dimers, then they bound to a GAG dp10 and, finally, MB dimers were further extended by π–π stacking interactions between benzene rings and stabilized via dye–GAG electrostatic interactions. Representative types of MB–GAG dp10 complexes are shown in Figure 4.

During the simulation of HP dp10 with MB, ligands interacted with the GAG during the whole 100 ns MD simulation forming two kind of stacks, containing four and six molecules. For the simulation with deHP dp10, there was no long lasting ionic stabilization of the established stacks by the GAG. During the whole MD simulation, the ligands associated with and dissociated from deHP dp10, forming complexes stabilized for 0.5–1.0 ns. It is to be noted that the carboxylate group in deHP interacted with the cationic MB molecules, but these interactions were not stable. For the simulations with CS4 and CS6, the largest conformational changes in the GAG chains were observed in relation to the other five simulations. Both CS4 and CS6 adopted a ligand-stabilized bent conformation due to interaction with MB. Additionally, the dye stack faced to the concave GAG surface, and sulfate groups were not in close contact with MB molecules. This could be explained by the lower net charge of CS in comparison to HS due to which the vdW interactions are more pronounced. In addition, this could be the reason for the observed conformational adaptation of the CS chains. MD simulations performed for three types of HS dp10 yielded similar results: dimeric stacks were stabilized by π–π interactions associating with GAGs dp10 and then formed oligomers containing 2–8 dye molecules. Among them, the most stable MB binding was observed for HS1 dp10, where the ligands remained bound to the same part of the GAG during the second half of MD simulation. In none of the MD simulations were stable dynamically restricted water molecules or Na^+^ counterions observed to participate in GAG–MB interfaces.

### 3.1. MM–GBSA Analysis

For all studied MB–GAG dp10 complexes, we performed MM–GBSA binding free energy analysis. The results are presented in Table 1, where all ∆G values are normalized with respect to the number of ligands interacting with GAGs dp10 in the corresponding MD simulation. 

The results for two unbound molecules show that the ∆G_eel_ value is unfavorable (44.6 kcal/mol), and drug–drug interactions are stabilized by van der Waals contacts. The ∆G_eel_ + ∆G_egb_ value is also slightly positive (1.5 kcal/mol), which further confirms the non-electrostatic nature of those interactions. Moreover, for the most of studied MB–GAG dp10 complexes, the ∆G_eel_ + ∆G_egb_ values are positive (MB–CS4 dp10, MB–CS6 dp10, MB–HS2 dp10). Electrostatic interactions are dominantly responsible for stabilization of the complexes only for HP dp10, HS1 dp10 and HS3 dp10, while the vdW contribution is the most favorable for CS6 dp10 (−50.5 kcal/mol) and HS2 dp10 (−55.2 kcal/mol). The least favorable values of the free energy of binding (∆G) per a drug molecule were observed for deHP dp10 (−0.3 kcal/mol) and HS3 dp10 (−2.0 kcal/mol) complexes. The higher relative contribution of Lennard-Jones interactions to the total energy in the case of CS and HS2 should not be misinterpreted. The interactions in all systems are electrostatically driven. However, in the case of less charged GAGs, the impact of the electrostatic term is lower and, therefore, the vdW free energy component, relatively, is more visible. At the same time, no specific vdW contacts between any particular MB and GAG moieties could be distinguished. The observed difference for HS1-3 was surprising since all three analyzed HS dp10 possess the same number of sulfates and, therefore, have the same net charge. This could suggest the dependence of MB recognition on the GAG sulfation pattern and may explain why molecules interact with HS3 dp10 only within short time intervals. All values of free energy of binding were very similar when comparing MM-GBSA results for MB–CS4 dp10 and MB–CS6 dp10 complexes.

### 3.2. The Sulfation Pattern Dependence 

In order to check if the number of MB molecules interacting with a GAG was dependent on its sulfation pattern, we compared the number of GAG binding dye molecules during MD simulation (Table 2) to the numbers of sulfate groups in the studied GAGs as follows

D = n_MB_ × t/n_SO3-_ (1)

where n_MB_ is the number of MB molecules interacting with GAG dp10 at the same time during MD simulation, n_SO3−_ is the number of sulfates in GAG dp10 and t is the fraction of the MD simulation time when n_MB_ interacts with GAGs. Such an analysis was made since according to recent UV spectrophotometric studies [35], the binding ability of MB to HS was independent of the sulfate position and basic structure of the saccharide ring.

When analyzing the sulfation dependence of GAG bound MB molecules, it should be taken into account that two types of GAGs were tested: HS and CS. For the HS, the advantage of the electrostatic component of the binding energy is clearly visible. The calculated D value is within 0.10–0.50 for HS. These oligosaccharides have fewer sulfate groups than HP, so the MB molecules bound in the same amount as to HP for a shorter interval, with the exception of the MB–HS2 complex. CS4 and CS6, compared to HP and HS, contain fewer sulfate groups, but this does not affect the amount of non-covalently bound drugs due to the stacks’ formation. This is reflected in the D values being higher for both CS than for HP and HS (0.60 and 1.00). It can, therefore, be concluded that for the CS, the number of interacting MB molecules is independent of the sulfation pattern.

### 3.3. MB–GAG Distance Analysis

**Figure 5:**
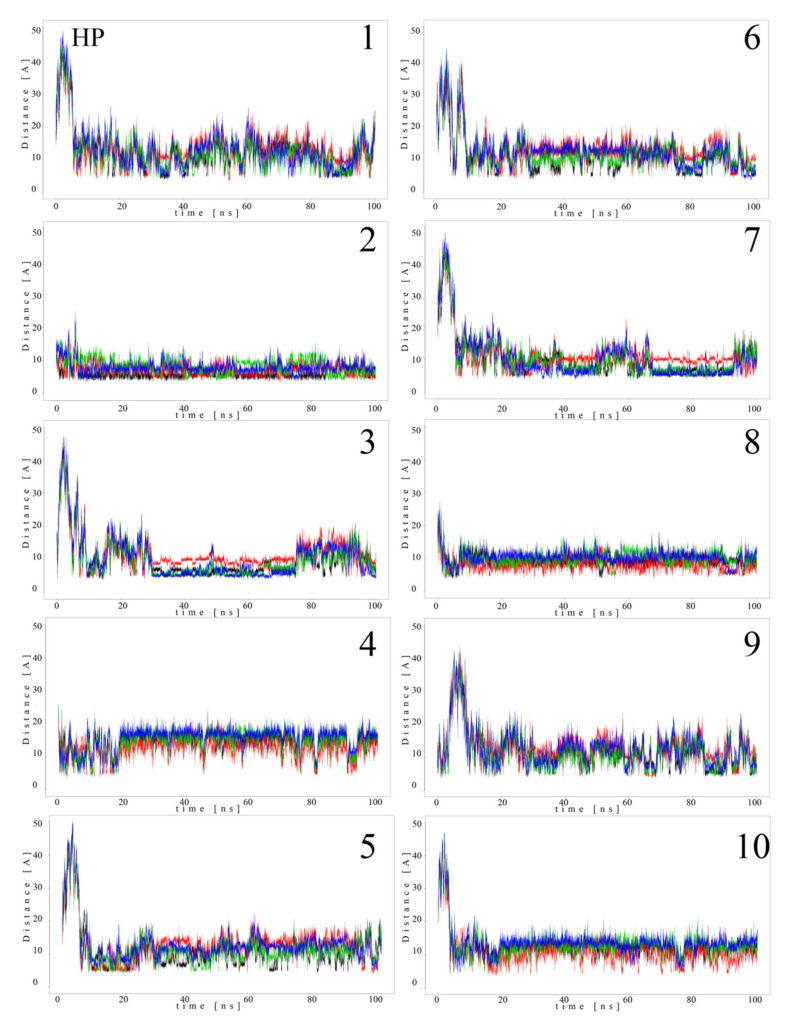
Distances (Å) between HP dp10 sulfate groups (corresponding sulfur atom), the carboxylate group (corresponding carbon atom) and one of the nitrogen atoms from the dimethylamino moieties of MB molecules (**1**–**10**) during MD (HP: black, glucosamine *N*-sulfates; blue, glucosamine 6-*O*-sulfates; red, iduronic acid 2-*O*-sulfates; green, carboxylate groups).

**Figure 6:**
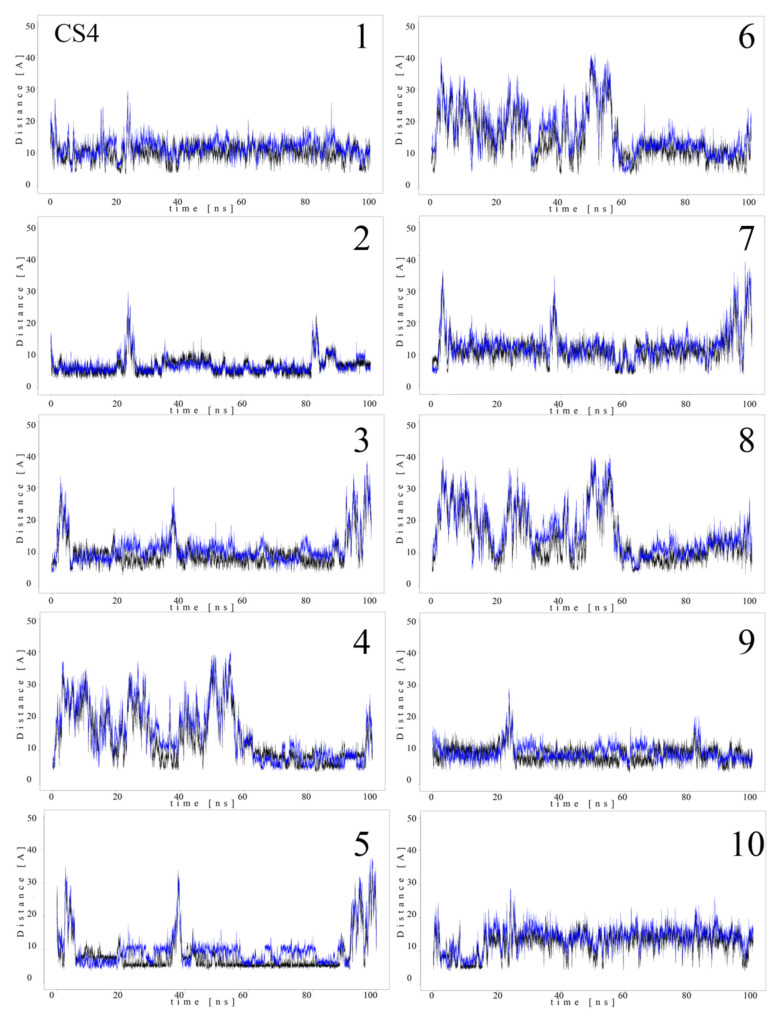
Distances (Å) between CS4 dp10 sulfate groups (corresponding sulfur atom), the carboxylate group (corresponding carbon atom) and one of the nitrogen atoms from the dimethylamino moieties of MB molecules (**1**–**10**) during MD (black, *N*-acetylgalactosamine-4-sulfates; blue, carboxylate groups).

Since MB molecules associated with and dissociated from GAGs dp10 during the MD simulations, we decided to conduct a detailed analysis of this process. Calculations of the distance between the GAG sulfate groups of different types, the carboxylate groups and one of the terminal nitrogen atoms of MB were performed. Figure 5 and Figure 6 show the obtained values for HP dp10 and CS4 dp10.

In the MB–HP dp10 complex, four MB molecules formed one stack and six molecules made another stack, and both were stable during the whole MD simulation (distance about 8 Å). Similarly, for the MB–CS4 dp10 complex, one stable dimeric stack was established, and its GAG interaction was observed after 60 ns. The remaining MB molecules bound to CS4 dp10 for shorter time intervals forming dimers and higher oligomers. In order to thoroughly analyze the results, the MD simulation fraction for each complex containing individual MB–sulfate and MB–carboxylate pairs were calculated (Table 3). The distances for the individual MB molecules as defined between sulfate and carboxyl groups of a GAG and the positively charged dimethylamino group of MB in the complexes were summed and the cutoff value was set at 8 Å. In the MB–CS4 complexes the sulfates were most often within a range that allows dye–GAG interaction (30.2% of MD simulation time). In turn, for deHP dp10 and HS3 dp10, the interactions between MB molecules and the carboxylate group of these GAGs decreased, especially for HS3 to 1.6% of the MD simulation time. The interactions between MB and the HS2 N-sulfate group as well as between MB and the HS3 6-sulfate group were essentially less populated than for other analyzed HS.

## 4. Conclusions

In this work, we applied MD-based approaches to evaluate molecular interactions between MB and GAGs dp10 expanding the knowledge obtained previously for other GAG–small molecule systems [13,14,52], and aiming to better understand experimental data available in the literature [35]. We performed and analyzed MD simulations for two and ten unbound MB molecules and for MB complexes of seven different GAGs dp10. Significant differences were found between HS and CS, reflected in the nature of the non-covalent interactions with MB. The interactions in MB–HP and MB–HS complexes are clearly electrostatically driven. The inherently helix-like structure of both HP and HS are restricted due to intrachain ionic repulsions between the sulfate groups and thus does not show any significant changes upon interaction with MB molecules. Distinctly from HP and HS, MB–CS complexes are rather stabilized by van der Waals contacts since the carboxylate groups do not establish as strong ionic contacts as sulfate groups with the terminal dimethylamino groups of MB. Due to the lower net charge of the disaccharide units, CS chains are somewhat more flexible than HP and HS but still more structured than the protein-like random coil. Accordingly, MB binding affects their conformations more, resulting in a slightly bent overall structure. We also analyzed the dependence of the number of bound MB molecules on the GAG sulfation degree. Jia and co-workers [35] showed that MB–HS binding is independent of the sulfate position and the anomeric state of uronic acid. Our data revealed a relationship between the number of sulfate groups, the number of bound dye molecules and their interaction time for MB–HP/HS complexes. The binding of MB to HP/HS is more stable when the GAG contains more sulfate groups, which is in contrast to the findings of Jia and co-workers, suggesting the increase in bound MB fraction as the sulfation content of HS decreased (see Supplementary Material Table S1). For HS with a lower sulfation degree than in HP, the binding of the same number of MB molecules is less stable (except for the MB–HS2 complex). However, by considering the dependence of the number of GAG binding drug molecules during MD simulation on the GAG sulfation degree, it is stronger for HS1 and HS3 than for the HP. On the other hand, this rule does not apply for the MB–CS adducts, where no such dependence was observed but rather distinct interactions related to the sulfation pattern. The novelty of the presented data consists of gained insights into the molecular mechanism of MB–GAG interactions, underlying the practical aspects of MB utilization as a colorimetric sensor for GAG quantification and pointing out GAG-specific differences that can be encountered during the employment of bioanalytical protocols. At the same time, the strong GAG–MB interactions could be considered for potential applications in which GAG serves as a carrier of MB and related drugs. These results increase our knowledge available so far about molecular aspects of small molecule–GAG interactions and provide new opportunities in drug design and potential therapies for diseases in which the GAG interactome is involved.

## Figures and Tables

**Table 1:** MM–GBSA binding free energy analysis of MB–GAG dp10 complexes.

System	Number of Drug Interacting	∆G	∆G/Drugs Number	∆G_vdW_	∆G_VdW_/Drugs Number	∆G_eel_	∆G_eel_/Drugs Number	∆G_esurf_	∆G_esurf_/Drug Number	∆G_eel_ + ∆G_egb_	∆G_eel_ + ∆G_egb_/Drugs Number
MB–MB	2	−13.0	−6.5	−15.1	−7.5	44.6	22.3	−1.0	−0.5	3.1	1.5
HP/MB	10	−55.0	−5.5	−44.6	−4.5	−3263.4	−326.3	−5.1	−0.5	−5.4	−0.5
deHP/MB	7	−2.8	−0.3	0.0	0.0	−174.1	−17.4	0.0	0.0	−2.1	−0.2
CS4/MB	10	−43.6	−4.4	−45.7	−4.6	−1679.2	−167.9	−4.2	−0.4	6.4	0.6
CS6/MB	10	−50.0	−5.0	−50.5	−5.1	−1861.6	−186.2	−5.0	−0.5	5.6	0.6
HS1/MB	10	−29.9	−3.0	−22.4	−2.2	−2209.2	−220.9	−3.1	−0.3	−4.5	−0.5
HS2/MB	10	−55.5	−5.6	−55.2	−5.5	−2322.3	−232.2	−5.4	−0.5	5.0	0.5
HS3/MB	10	−20.3	−2.0	−11.3	−1.1	−1639.1	−163.9	−1.4	−0.1	−7.6	−0.8

∆G, ∆G_vdW_, ∆G_eel_, ∆G_esurf_, ∆G_egb_ are MM–GBSA full binding free energy, van der Waals, *in vacuo* electrostatic, non-polar solvation, Generalized Born reaction field components, respectively. All values are in kcal/mol.

**Table 2:** Dependence of the number of dye molecules interacting simultaneously with GAGs during MD simulation on the number of sulfate groups in the GAGs under study.

Lp	GAG dp10	n_MB_	n_SO_^3−^	t	D
1	HP	10	15	0.42	0.28
2	deHP	7	0	-	-
3	CS4	10	5	0.30	0.60
4	CS6	10	5	0.50	1.00
5	HS1	10	10	0.30	0.30
6	HS2	10	10	0.50	0.50
7	HS3	10	10	0.10	0.10

**Table 3:** Distance analysis for interactions between MB and GAGs expressed in terms of the MD simulation time fractions.

Lp	GAG dp10	% of the Low Distance * with *N*-Sulfates	% of the Low Distance * with 4-Sulfates	% of the Low Distance * with 6-Sulfates	% of the Low Distance * with 2-Sulfates	% of the Low Distance * with Carboxylate Group
1	HP	29.7	-	20.3	22.0	23.6
2	deHP	-	-	-	-	13.4
3	CS4	-	30.2	-	-	21.1
4	CS6	-	-	28.4	-	29.1
5	HS1	29.9	-	-	31.5	25.9
6	HS2	19.3	-	31.6	-	30.1
7	HS3	-	-	28.4	23.0	1.6

* Low distance—distance between one of the nitrogen atoms from the dimethylamino moieties of MB and a group from a GAG dp10 below 8 Å.

## Data Availability

Not applicable.

## Supplementary Materials

The following supporting information can be downloaded at https://www.mdpi.com/article/10.3390/molecules27092654/s1, Figure S1: Density of probability for the distance between the planes defined by the aromatic carbons of two MB molecules in a stack for the: (A) two unbound MB and (B) MB-HP dp10; Table S1: Comparison of the binding free energies in MB-GAG dp10 complexes analyzed in the present study to the previously obtained binding ratios by Jia et al. [35].
