# Supplementary material for: Molecular Dynamics Approaches Dissect Molecular Mechanisms Underlying Methylene Blue–Glycosaminoglycan Interactions
Source: Molecules. 2022 Apr 20;27(9):2654. doi: 10.3390/molecules27092654 (PMC9105714; doi:10.3390/molecules27092654)
Supplement: Supplementary file 1 [file molecules-27-02654-s001.zip › molecules-1671296-supplementary.pdf]

molecules

Supplementary Materials

Molecular Dynamics Approaches Dissect Molecular Mechanisms Underlying Methylene Blue–Glycosaminoglycan Interactions

Martyna Maszota-Zieleniak [1], Ferenc Zsila [2] and Sergey A. Samsonov [1,*]

[1] Faculty of Chemistry, University of Gdańsk, ul. Wita Stwosza, 63, 80-308 Gdańsk, Poland; m.maszota.zieleniak@gmail.com
[2] Institute of Materials and Environmental Chemistry, Research Centre for Natural Sciences, Hungarian Academy of Sciences, Magyar Tudósok Körútja 2, 1117 Budapest, Hungary; zsila.ferenc@ttk.hu
* Correspondence: sergey.samsonov@ug.edu.pl; Tel.: +48-58-523-5166

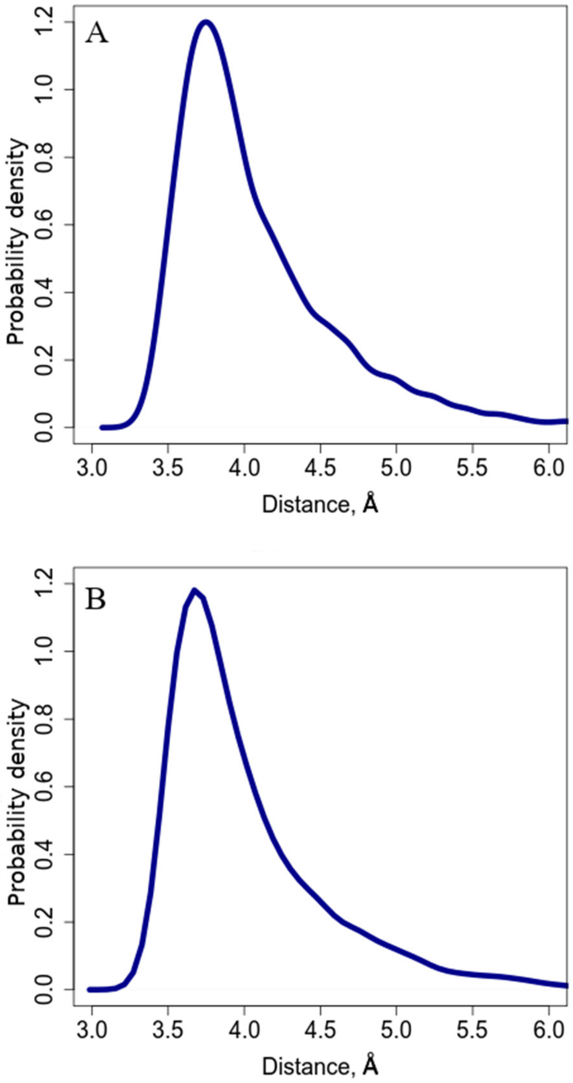

Figure S1. Density of probability for the distance between the planes defined by the aromatic carbons of two MB molecules in a stack for the: A) two unbound MB and B) MB-HP dp10.

Table S1. Comparison of the binding free energies in MB-GAG dp10 complexes analyzed in the present study to the previously obtained binding ratios by Jia et al. [35].

GAG dp10	ΔG/drugs number[*]	Binding ratio [35][**]
deHP	+	-
HP	+++	+
HS1	+	+++
HS2	+++	++
HS3	+	+++
CS4	++	-
CS6	+++	-

[*] +: $\Delta G < 0$ kcal/mol; ++: -5 kcal/mol $< \Delta G < -3$ kcal/mol; +++: $\Delta G < -5$ kcal/mol;. [**] + binding ratio > 0; ++ $3 <$ binding ratio < 4; +++ binding ratio > 4 (higher binding ratio corresponds to stronger binding).
